# Automated multimodal spectral histopathology for quantitative diagnosis of residual tumour during basal cell carcinoma surgery

**DOI:** 10.1364/BOE.8.005749

**Published:** 2017-11-22

**Authors:** Radu Boitor, Kenny Kong, Dustin Shipp, Sandeep Varma, Alexey Koloydenko, Kusum Kulkarni, Somaia Elsheikh, Tom Bakker Schut, Peter Caspers, Gerwin Puppels, Martin van der Wolf, Elena Sokolova, T. E. C. Nijsten, Brogan Salence, Hywel Williams, Ioan Notingher

**Affiliations:** 1 School of Physics and Astronomy, University Park, University of Nottingham, Nottingham, NG7 2RD, UK; 2 Circle Nottingham Ltd NHS Treatment Centre, Lister Road, Nottingham NG7 2FT, UK; 3 Mathematics Department, Royal Holloway University of London, Egham, TW20 OEX, UK; 4 Department of Pathology, Nottingham University Hospitals NHS Trust, QMC Campus, Derby Road, Nottingham, NG7 2UH, UK; 5 Erasmus-University Medical Center Rotterdam, Department of Dermatology, The Netherlands; 6 RiverD International, Marconistraat 16, Rotterdam 3029 AK, The Netherlands; 7 East Surrey Hospital, Canada Ave, Redhill RH1 5RH, UK; 8 Centre of Evidence-Based Dermatology, Nottingham University Hospital NHS Trust, QMC Campus, Derby Road, NG7 2UH, UK

**Keywords:** (170.0170) Medical optics and biotechnology, (170.3880) Medical and biological imaging, (300.6450) Spectroscopy, Raman

## Abstract

Multimodal spectral histopathology (MSH), an optical technique combining tissue auto-fluorescence (AF) imaging and Raman micro-spectroscopy (RMS), was previously proposed for detection of residual basal cell carcinoma (BCC) at the surface of surgically-resected skin tissue. Here we report the development of a fully-automated prototype instrument based on MSH designed to be used in the clinic and operated by a non-specialist spectroscopy user. The algorithms for the AF image processing and Raman spectroscopy classification had been first optimised on a manually-operated laboratory instrument and then validated on the automated prototype using skin samples from independent patients. We present results on a range of skin samples excised during Mohs micrographic surgery, and demonstrate consistent diagnosis obtained in repeat test measurement, in agreement with the reference histopathology diagnosis. We also show that the prototype instrument can be operated by clinical users (a skin surgeon and a core medical trainee, after only 1-8 hours of training) to obtain consistent results in agreement with histopathology. The development of the new automated prototype and demonstration of inter-instrument transferability of the diagnosis models are important steps on the clinical translation path: it allows the testing of the MSH technology in a relevant clinical environment in order to evaluate its performance on a sufficiently large number of patients.

## 1. Introduction

Basal cell carcinoma (BCC) is the most common cancer type in humans. More than 100,000 cases of BCC are diagnosed each year in the UK (1.7 million in the USA, and 300,000 in Australia) [1]. Patients expect their BCCs to be treated effectively in a single operation, with minimal risk of tumor recurrence, and the best cosmetic result possible. Most BCCs (>80%) occur on the head and neck areas, in particular the upper central part of the face [2]. The majority of these BCCs are treated by wide-local excision performed under local anesthesia in an outpatient setting. The treatment outcome of wide-local excision depends on the characteristics of the tumor (subtype, location), as well as how well the surgeon can identify its spread. For example, a 4 mm surgical margin for primary well-defined BCC measuring less than 20mm ensures a complete clearance of over 95% [3] while a 3mm margin, even for lesions that measure 6 × 5 mm, will clear only about 85% of tumors [4,5]. Large BCCs (>2cm) and those occurring at high risk sites (nose, ear, eyelid, eyebrow, and temple) are at higher risk of incomplete treatment, and Mohs micrographic surgery is preferred [6–8]. In Mohs surgery, sequential layers of tissue are excised and microscopically examined as frozen sections to make sure that all cancer is removed; if residual cancer is present, then the exact location of those cells is recorded and further layers of skin tissue are removed until margins are clear. Although wide-local excision is the most common surgical technique for treating BCC, histopathological confirmation of complete removal takes 1-2 weeks. Incomplete removal requires specialist follow-up or a second operation to remove the residual cancer (typically by Mohs surgery), which can lead to patient anxiety and poorer cosmetic outcome, especially if a skin graft from the initial treatment needs to be redone. Although Mohs surgery is typically recommended for high-risk BCCs [9], this complex surgery requires specialist surgeon and treatment facilities, and is labor intensive, needing dedicated technicians to prepare frozen tissue sections.

Several technologies have been developed recently to allow microscopic assessment of tumor clearance during BCC surgery, including confocal microscopy (CM) [10,11], optical coherence tomography (OCT) [12,13] and Raman micro-spectroscopy [14]. While CM and OCT enable diagnosis of BCC based on structural features, Raman spectroscopy can measure endogenous molecular differences between healthy skin tissue and BCC, with high chemical specificity [14–18]. Therefore, Raman spectroscopy offers the prospect of objective diagnosis based on quantitative molecular analysis of tissue, which has the potential to reduce intra- and inter-user variability, as well as diagnosis subjectivity [19, 20]. Although Raman spectroscopy is typically slow to allow imaging of cm-scale tissue samples within time-scales compatible with intra-operative use (e.g. <1 hour), selective-sampling Raman spectroscopy techniques have been recently developed to reduce measurement time [14, 21]. Our team has developed a multimodal spectral imaging technique that integrates tissue auto-fluorescence imaging and Raman microscopy that can be used during tissue conserving surgery for fast and objective detection of residual tumor [14, 22–24]. Using laboratory instruments, that partially included input from user (manual focusing and change of objectives when switching between auto-fluorescence and Raman modalities), previous studies demonstrated the feasibility to obtain quantitative diagnosis images based on molecular analysis of tissue combined with multivariate classification models.

Here, we report the development of a fully-automated prototype instrument based on the combined auto-fluorescence (AF) and Raman micro-spectroscopy (RMS) multimodal approach, that allows fully automated measurements of skin surgical resections and objective diagnosis of residual BCC. The instrument has been designed and built to fit into clinical practice as an alternative to current practice of microscopic histological assessment of frozen tissue sections for checking the completeness of BCC removal during all BCC surgery. First, we show that the optimized algorithms for analysis of the AF images and Raman classification models developed on a laboratory instrument can be successfully transferred on the fully automated new device. This is an important step because inter-instrument transferability remains one of the key challenges in the clinical translation of Raman spectroscopy. Secondly, we present a model for establishing a threshold based the MSH diagnosis image that allows a binary diagnosis at a tissue sample level: margins clear of BCC detected: Yes/No. Finally, we investigated the consistency and validity of the MSH diagnosis by comparing the MSH assessment output in repeat test measurements recorded by a spectroscopy specialist user and clinical users (Mohs surgeon and medical trainee), on a range of skin samples excised during Mohs micrographic surgery, and using histopathology as the standard of reference.

## 2. Methods

### 2.1 Tissue samples

Skin tissue samples were obtained during Mohs micrographic surgery at the Nottingham University Hospitals National Health Service (NHS) Trust and the Nottingham NHS Treatment Centre. Ethical approval was granted by the Nottingham Research Ethics Committee (07/H0408/172) and informed consent was obtained from all patients. After excision by the surgeon, the tissue layer was embedded within optimal cutting temperature medium (OCT) and frozen with a cryogenic spray (Frostbite, Surgipath). For each tissue sample, a 10 μm section (called adjacent section) was cut and stained by hematoxilin and eosin (H&E) and used as the standard of reference. The remaining tissue block was then defrosted, washed and kept frozen at −20°C until used for Raman spectral measurements.

In this study, samples from 80 patients with basal cell carcinoma on the face and neck were included. The sample set was divided into a training set (50 samples, 30 patients) that was measured on the Laboratory Instrument in order to train and optimize the diagnosis algorithms. All the samples in the training set were used for the training of the Raman classification model, but only the BCC positive samples (35 samples, 23 patients) were used for optimization of the algorithms for AF image segmentation and sampling points generation. The test set consisted of 47 samples from new 40 patients, which were measured on the Prototype instrument to determine the performance of the algorithms. As with the samples recorded with the Laboratory Instrument, all test samples were used in the testing of Raman classification model, while only the BCC positive samples were used to validate the AF algorithms (32 samples, 27 patients). Then, 10 samples (9 patients, no overlap with the training or test sets) were used to test the overall performance of the MSH diagnosis on the Prototype device, including illustration of intra- and inter-user repeatability.

### 2.2 Instrumentation

#### *Laboratory Instrument*.

The instrument consisted of an inverted optical microscope (Eclipse-Ti Nikon) with an automated sample stage (H107 with Proscan II controller, Prior Scientific), 60 × /1.2NA objective (RiverD International), 785nm laser (IPS, power 100 mW at sample surface), spectrometer (RiverD International HPR) equipped with a deep-depletion back-illuminated cooled CCD (DU401-A-BR-DD, Andor Technology). The acquisition time for the Raman measurements ranged between 0.5 and 3 seconds per position.

#### *Prototype instrument*.

**Fig. 1:**
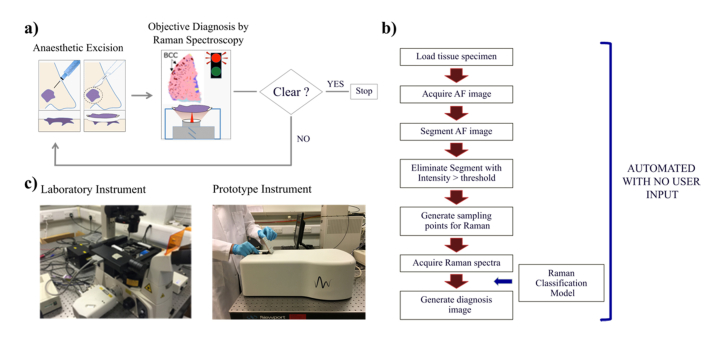
a). Schematic description of the intended use of MSH for checking completeness of tumour removal during BCC surgery (Mohs surgery and wide-local excision). b) Flow chart describing the automated measurement and diagnosis algorithms for MSH. c) Photographs of the manual Laboratory Instrument and the automated MSH Prototype. The tissue specimen is loaded in a purpose-built cassette with a quartz bottom window (2.5mm x 2.5cm, 1mm thick). The cassette is manually placed on the microscope stage of the Laboratory Instrument or loaded into the Prototype Instrument.

The instrument was developed jointly by University of Nottingham and RiverD International. The requirements and specifications for the clinical application were established through consultation with 3 general dermatologists, 23 skin surgeons (including Mohs and plastic surgeons) and 4 histopathologists. Its operation at all analysis steps was fully automated (see Fig. 1(b), 1(c)

). The instrument was designed to analyse skin specimens of up to 2cm × 2cm in size (thickness up to 5mm), which would accommodate the whole excision surface of most resection specimens in Mohs surgery (including simultaneous analysis of up to 4 skin samples fitting the 2cm × 2cm area). The samples used in this study represented halves or quarters of spare skin samples resected during Mohs surgery. The samples were placed in purpose-designed disposable cassettes. After loading of the cassette in the instrument, a colour image of the sample is displayed on the screen to confirm the correct position of the tissue samples and to allow the user to identify the red and blue dye markings used for orientation of the tissue with respect to the surgical opening. The software included automated calibration, quality control checks and autofocusing of the Raman measurements. After the input of the required patient data, the user can start the analysis of the tissue by selecting the “Start” button. No additional input is required from the user to reach the final diagnosis. The software provides real time display of the AF images and presents the final MSH diagnosis as a colour-coded image.

### 2.3 Data post-processing and analysis

#### *Auto-fluorescence imaging*:

Auto-fluorescence images of the tissue samples were acquired by stitching 16 image tiles (6.4mm x 6.4mm) to create a 2cm × 2cm image. The region of the image containing tissue was identified automatically and separated from the background by using a set intensity threshold. The AF image was then flattened with a rotationally symmetric Gaussian low pass filter to minimize fluctuations in intensity and stitching artefacts. The segmentation of the resulting AF image was then performed using an intensity thresholding based on the optimization described in Section 3.

#### Raman spectroscopy.

All Raman spectra were corrected for cosmic rays and were calibrated using a neon-argon light source and a PMMA sample, and corrected for the wavelength dependent sensitivity of the set up using a NIST calibration standard. Background signals from optical elements in the instrument were subtracted from the spectra. Spectra were then interpolated in the range of 400-1800 cm^−1^ to 2 cm^−1^ data spacing. For the training data set, raster scans were performed on the surface of the removed tissue samples, adjacent to the H&E section. Spectra were acquired with a 0.5 s exposure time and were averaged with a 3x3 moving average filter. Spectra were utilized in the classification model only if their signal to noise ratio (SNR) was above 15, where SNR was calculated as the ratio between the height of the CH_2_ peak at 1450 cm^−1^ (local linear baseline subtracted) and *rms* value of the noise in the empty spectral region 1750-1800 cm^−1^. For the test set, the Raman spectra were acquired with the Prototype instrument with a modified version of the MSH procedure that allowed acquisition of a larger number of spectra for each sample. To maximize the number of Raman spectra to be included in the test set, the minimum number of spectra per segment was increased to 20 and the total number of spectra acquired per tissue sample was limited to 1200. The integration time was set to 3 s per spectrum and spectra with SNR lower than 7 were discarded. Spectral features (areas of Raman bands) were calculated from the Raman spectra of both training and test samples using a local linear background subtraction for each band, and were then the normalized to unit norm on a per spectrum basis.

### 2.4 MSH diagnosis

The MSH algorithm generated the diagnosis of each segment obtained by the AF segmentation algorithm independently using the Raman spectra measured inside the segment in an automated two-step process. In the first round, Raman spectra were measured at the locations determined by the sampling point generation algorithm (spectra with SNR lower than 4 were discarded). More sampling points were generated to allow new Raman spectra to be measured in the second stage of the MSH procedure, to replace the spectra that are discarded. Segments for which more than 80% of the spectra were discarded, were labelled “Unclassified” (no more spectra were measured for these segments in the second round). The retained spectra were denoised based on an independent set of Raman spectra (100,000 spectra collected from samples in the training set that were not included in the training set because the SNR was between 10 and 15) using principal component analysis (PCA) with 50 PCs [25]. Then, each Raman spectrum was classified by applying the Raman classification model. If the segment contained no spectra classified as BCC and the class of all spectra was the same, the segment was labelled accordingly. In cases in which the segment contained spectra from more than one class (but no BCC) within a segment, a nearest neighbour evaluation was performed within the segment for each of the sampling points and the segment was split into regions of nearest proximity for each sampling point location, as described previously [22]. If the segment contained only one BCC spectrum, the spectrum was ignored and the segment was labelled as above. If more than 80% of spectra were classified as BCC, the segment was labelled as BCC. If a segment had at least two spectra classified as BCC, but this accounted to less than 80% of the total number of spectra in the segment, a second round of Raman spectra were acquired for the segment. For each segment included in the second round, the number of sampling points was equal to the number in the first round, and were uniformly distributed in the segment (the locations of the first round were taken into account to avoid measurements at the same locations). The spectra in the second round were retained only if they pass the SNR threshold. The Raman spectra obtained from the two rounds of measurements were then joined and were classified using the Raman classification model. The results of the classification model were then interpreted on a per spectrum basis and the final labelling of each segment was performed using the nearest neighbor method described above. The final MSH diagnosis image was generated using a color code for each class.

## 3. Results

MSH provides molecular analysis of skin tissue specimens excised during BCC surgery without requiring any additional preparation steps. The aim is to obtain an image of the tissue that highlights the residual BCC at the surface of the resected specimen, using color-coded labels that indicate the tumour (Fig. 1(a)). Based on this image, the surgeon can make an objective decision whether to excise further tissue at the location indicated by the color-coded image or whether to stop the surgery.

### 3.1 Optimisation of the measurement and diagnosis algorithms based on the laboratory Instrument

In order to achieve reliable and objective diagnosis of BCC, the complete workflow (Fig. 1(b)) must be invariant across the range of samples obtained in BCC surgery, and be transferable between instruments. In this way, once the algorithms have been optimised on one device, they can be transferred to all devices deployed in clinics. Thus, every step in the diagnosis workflow described in Fig. 1(b) was first optimised using a training set of samples analysed on the Laboratory instrument, for which the user was able to manually adjust the focusing and changing the objectives when switching between the AF and Raman measurements (Fig. 1(c) left). The algorithms were then tested using a new set of independent skin samples analysed on a fully automated Prototype, for which the user operation required only the loading of the tissue specimen.

#### 3.1.1 Segmentation algorithms for the auto-fluorescence imaging

After the acquisition of the AF image of a skin sample, the entire image was segmented using an intensity threshold. However, because the AF images exhibit large inter-sample variations in intensity (caused by inter-patient variations in tissue depending on age, anatomic location, or pathology, potential laser intensity fluctuations), we developed a user-independent algorithm that optimises the value of the intensity threshold for each individual image. To quantify the performance of this algorithm, two metrics were defined:

$$\rho =\frac{Area(detected\;BCC)}{Area\left(detected\;BCC\right)+Area(missed\;BCC)}$$

This metric was calculated for all BCC positive samples in order to measure the fraction of the tumour detected and retained by the segmented AF image.

$$\varepsilon =\frac{Area\;(Healthy\;tissue)}{Area(detected\;BCC)+Area(Healthy\;tissue)}$$

The metric *ε* was calculated for each segment in an AF image containing BCC in order to estimate the histological heterogeneity of the segments. The values of *Area(detected BCC)*, *Area(missed BCC)* and *Area(Healthy tissue)* were obtained by comparing the generated segmentation maps with the AF annotated images (manually marking the BCC regions on the AF images based on the adjacent H&E sections). Based on a set of comparable adjacent H&E sections, we estimate that the use of adjacent H&E sections for the annotation can lead to errors of ~10-100μm because of differences in tumour morphology between the tissue analysed by AF and the H&E sections. Thus, ρ and ε are not an absolute measure of the performance of the segmentation algorithm to detect BCC, but rather offer an indication of how different methods of segmentation perform comparatively.

**Fig. 2:**
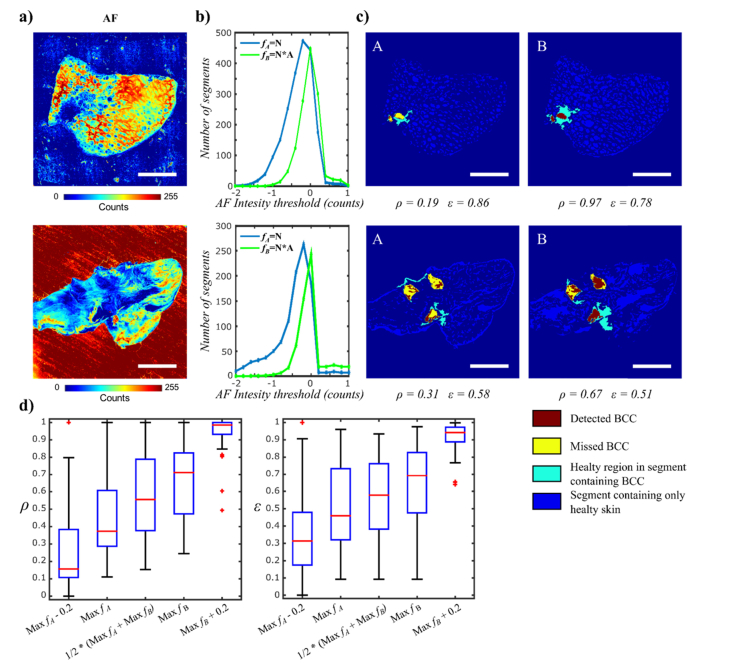
Optimisation and evaluation of segmentation algorithms for AF images of skin samples (recorded on Laboratory Instrument). a)-c) two typical examples: a) AF images; b) segmentation optimisation functions: *f_A_* = *N*, where *N* = segment number; *f_B_* = N*A, where *A* = area fraction captured by segments; c) segmentation images and quality parameters corresponding to the AF when using the *Maxf_A_* (A) and *Maxf_B_* (B) as intensity thresholds. Scale bars: 5mm. d) Statistical analysis of parameters ρ and ε based on AF images recorded on the Laboratory Instrument on 35 tissue samples with BCC (23 patients).

For an ideal segmentation algorithm, the entire tumour is retained for Raman spectroscopy analysis, *Area(missedBCC)* = 0, equivalent to ρ=1. In addition, all segments containing BCC would not contain any healthy tissue, *Area(Healthy)* = 0, equivalent to ε=0. Figure 2

presents some examples of segmentation results for typical skin tissue samples containing infiltrative and micro-nodular BCC. Figure 2(a) shows that the tumour regions have lower AF intensity compared to the stroma. Two functions were proposed for providing optimal segmentation thresholds invariant to the absolute intensity of the AF images: *f_A_ = N*, where *N* is the number of segments in the segmented image, f_B_ = N*A, where *A* is area fraction captured by the segments in the image. Figure 2(b) shows that both functions *f_A_* and *f_B_* have a global maximum when plotted as a function of the intensity threshold. Figure 2(c) presents the segmented AF images when the threshold used corresponded to the maximum values of functions *f_A_* and *f_B_*. For both samples, using the threshold value *f_B_* leads to a higher value of both ρ and ε compared to when using the *f_A_* value. These results indicate that *f_B_* increases the fraction of tumour detected, but also increases the histological heterogeneity of the segment (i.e. the amount of healthy tissue within the segments containing BCC also increased).

The statistical analysis of the segmentation parameters ρ and ε, obtained when using different thresholds for AF images of all BCC positive samples in the training set is presented in Fig. 2(d). The results show that using the maximum of the function *f_B_* (*Maxf_B_*) for selecting the optimal intensity threshold for segmentation provides a high detection rate for BCC (ρ= 0.3-1, median value 0.7), while ε ranged between 0.1 and 0.9 (median 0.7). Using the maximum of the function *f_A_* (*Maxf_A_*) provided slightly lower values of ε (ε = 0.1-1, median value 0.5), but the value of ρ also decreased to a median value of 0.35 (range of 0-0.9). Because from a clinical point of view the sensitivity is more important than specificity, the segmentation algorithm was set to use the threshold value generated by the function *f_b_* as it increased the capture probability for BCC.

Once the segmentation method was fixed, we evaluated the performance of the algorithm for generating sampling points for Raman spectroscopy measurements within the segments of the AF images. We employed a method that allocates a minimum number *N*_min_ of sampling points for all segments, with additional sampling points allocated to segments based on their area and variance in AF intensity [22]:

$$N_{sampling}\left(i\right)=N_{min}+\frac{Var(i)\times Area(i)}{\sum_{i=1}^{k}\left[Var(i)\times Area(i)\right]}\times N_{rest}$$

where $N_{sampling}\left(i\right)$ is the total number of sampling points allocated to the segment *i*, *N*_min_ is the minimum number of sampling points for each segment, *k* is the total number of segments, *Var(i)* is the intensity variance in the segment *i*, *Area(i)* is the area of the segment *i*, and *N_rest_* = *N_sampling, total_* –*k* × *N*_min_ is the additional sampling points that can be allocated to segments after the allocation of the minimum number of sampling points required for each segment. In this study, we used *N*_min_ = 5 and *N*_sampling total_ = 800. Using this method for allocating the number of sampling points *N_sampling_(i)*, two methods were investigated for determining the locations of the sampling points within each segment of the image. The first method placed sampling points at the locations with the lowest and highest intensity in the segment, followed by uniform distribution of the remaining sampling points (Configuration 1). The second method generated the sampling points uniformly within the segments (Configuration 2). Then, for each sample containing BCC, the tumour “Hit rate” was calculated as the ratio of the number of sampling points hitting BCC to the total number of sampling points allocated to segments containing BCC.

Figure 3

**Fig. 3:**
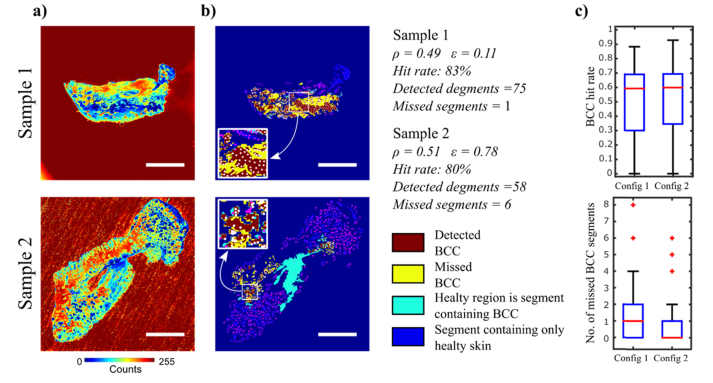
Evaluation of sampling points for Raman spectroscopy based on segmentation of AF images (Config. 2). Sample 1: typical sample containing BCC, Sample 2: the sample with the highest number of segments with BCC missed by the allocated sampling points. a) AF images; b) segmented images and sampling points using uniform distribution: pink points represent Raman measurements performed on healthy tissue and white points represent Raman measurements performed on BCC. The number of segments containing BCC detected and missed are indicated for each sample; Scale bars: 5mm. c) evaluation of tumour “Hit rate” and missed segments for: sampling points allocated selectively: sampling point at the position of the lowest and highest intensity within each segment, and uniform distribution of the remaining sampling points (Config. 1); sampling points distributed uniformly within segments (Config. 2).

presents examples of two skin samples containing BCC and the statistical analysis for all BCC positive samples in the training set. Figure 3(a), 3(b) show that ρ was ~0.50 for both samples, and the BCC regions were effectively detected in both samples: 75 and 58 segments containing BCC were detected in Sample 1 and Sample 2 respectively, and 80-83% of the sampling points within these segments coincided with the location of the tumour. Sample 2 was the sample with the highest number of missed segments with BCC in the entire training set (8 in Config. 1 and 6 in Config. 2). Figure 3(c) presents the statistical analysis of the BCC “Hit rate” and number of missed BCC segment for all tissue samples in the training set. The results show that both methods for generating locations of sampling points yield similar values for the BCC “hit rate” (0-0.9, median 0.6). Nevertheless, allocating sampling points uniformly within each segment provided a lower number of segments in which BCC regions were missed (0-2, median 0) compared to Configuration 1 (0-4, median 1). Based on this results, Config. 2 was selected for the final MSH algorithm.

#### 3.1.2 Classification model for BCC based on the Raman spectroscopy measurements

After completing the method of generating the sampling locations for Raman spectroscopy, MSH requires a classification method whereby each location in the tissue is classified (BCC or non-BCC) according to the Raman spectrum measured at the corresponding location. The first step for the Raman spectral classification model is the development of a database of Raman spectra measured by raster scanning and annotated using histology as a standard of reference. The Raman raster scan data were first analyzed by k-means clustering, which groups spectra into regions with similar spectra, allocating each spectrum to the cluster with the nearest mean and as such performs a rough delineation between groups of spectra with different spectral features, creating a 2D image of the investigated tissue surface.

**Fig. 4:**
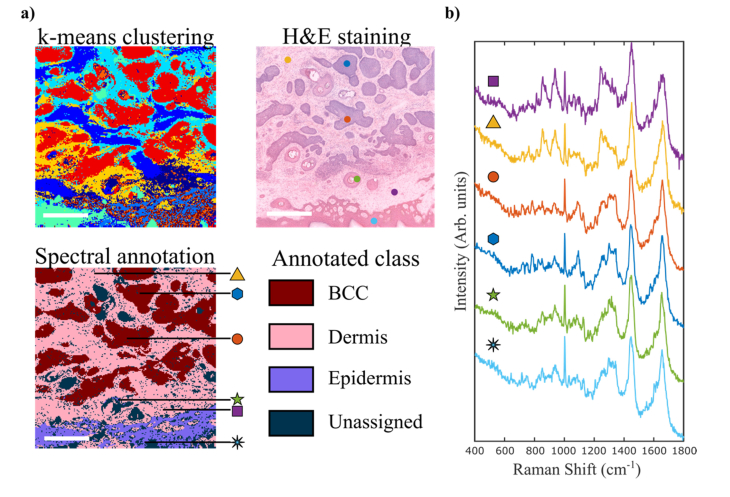
Development of the Raman classification model for BCC. a) annotation of Raman spectra based on histopathology following k means clustering of Raman spectra collected by raster scanning; Scale bar: 0.5mm. b) Selected Raman spectra at the location indicated by arrows and coloured coded symbols in the annotated Raman map and circles in the adjacent H&E image.

K-means clustering can be controlled by selecting the number of clusters it generates. The k- means image that is generated can be compared to the adjacent histopathological section and can be used to annotate a large number of spectra with the correct tissue class (Fig. 4(a)

). Figure 4(b) presents typical Raman spectra of skin tissue (measurement locations in the tissue indicated by arrows), which contained typical spectral features of skin tissue (Raman bands assigned to proteins, nucleic acids and lipids) [19].

**Fig. 5:**
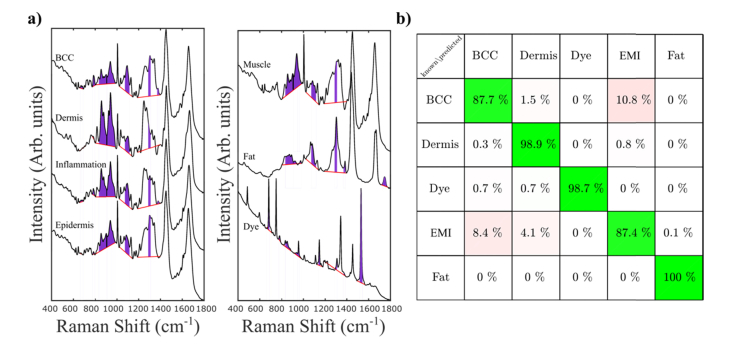
Development of the Raman classification model for BCC. a) Average Raman spectra of BCC, healthy skin tissue structures and surgical dye. The spectral features selected for the ANN classifier are highlighted (red lines represent the local baseline); b) confusion matrix for the 5-fold cross validation on the training set of samples measured on the Laboratory instrument. Class EMI includes epidermis, muscle and inflammation.

The Raman spectra of BCC were characterized by intense bands corresponding to DNA (788 cm^−1^, 1098 cm^−1^) [18] compared with other tissue structures. The Raman spectra of dermis were dominated by the bands assigned to collagen at 851 and 950 cm^−1^ and by the Amide III band [18]). Following this procedure, the Laboratory instrument was utilized to scan 50 tissue samples from 30 patients, which were then processed and used in the construction of the spectral classifier. In order to obtain a diagnosis model for BCC, several Raman feature selection and classification models were developed, and their performance estimated first by 5-fold cross-validation. Although it is common to use principal component analysis (PCA) for feature selection, PCA tends to also include small spectral features that often can be at the same level or lower than variations between instruments. Because our aim was to transfer the Raman classification model from the Laboratory Instrument on the Prototype device, we decided to manually select spectral features (areas of Raman bands) that could be reliably measured and showed differences between classes larger than inter-instrument variations. The highest performance for BCC detection was obtained when utilizing an Artificial Neural Network with 20 nodes in a single hidden layer with 13 spectral features. The average Raman spectra of the main tissue types included in the classification models are presented in Fig. 5(a)

: BCC, dermis, inflammation, epidermis (including hair follicles), muscle, fat (including sebaceous glands). The dye used by surgeons to mark the edges of the tissue can be easily identified by the several specific Raman bands at 680 cm^−1^, 748 cm^−1^, 1144 cm^−1^ and 1528 cm^−1^. Given the spectral similarities between the Raman spectra of epidermis, muscle and inflammation, these spectra were grouped into a single class (labelled “EMI”) in order to simplify the classification model.

The spectral features used as the input for the classification models were calculated as the areas of selected Raman bands that provided stable classification of BCC: 675 cm^−1^, 680 cm^−1^, 786 cm^−1^, 870 cm^−1^, 906 cm^−1^, 944 cm^−1^, 952 cm^−1^, 1092 cm^−1^, 1144 cm^−1^, 1298 cm^−1^, 1376 cm^−1^, 1528 cm^−1^, 1744 cm^−1^. The confusion matrix obtained by 5-fold cross-validation (Fig. 5(b)) indicates that BCC can be discriminated from all other tissue types with overall 87.7% sensitivity and 98.4% specificity. Whilst the classification model obtained > 98.7% sensitivity and specificity for dermis and fat, it correctly distinguished BCC from the EMI class (epidermis, inflammation and muscle) in ~89.2% of spectra.

#### 3.1.3 Criterion for MSH diagnosis at sample level

Based on the estimated performance of the AF imaging analysis algorithms and the Raman spectroscopy classifier, as well as on certain simplifying assumptions (see below), we investigated the statistically optimal criterion (uniformly most powerful test) for deciding if a sample is BCC positive or negative. Namely, given a threshold *N_th_* on the number *N_BCC_* of BCC-labeled segments in the MSH diagnosis image, the criterion is to call the sample “BCC-positive” if *N_BCC_*≥*N_th_*, and “BCC-negative” otherwise (i.e. if *N_BCC_*<*N_th_*). To derive the necessary expressions we simplified the two-round rule for labelling a segment as follows: a segment is labelled as BCC if and only if at least two spectra are (individually) classified as BCC. Similarly, we assumed the number of Raman spectra per segment *N_sampling_(i)* to be the same for all segments and equal to *N*_min_ *= 5*.

Estimating the “hit rate” as 0.6 and the per spectrum sensitivity and specificity as 81.8% and 96.3%, respectively, we obtained the predicted per segment 81.94% sensitivity and 98.73% specificity. Using the number of non-BCC segments in the sample *N_sampling,nonBCC_ = 150*, and the above per segment specificity estimate, the corresponding per sample specificity estimates was 95.65% when *N_th_* = 5. Using the above per segment sensitivity estimate and specifying, and considering the worst case scenario when the samples has single BCC segment (*N_sampling,BCC_ = 1)*, the corresponding per sample sensitivity was 11.03% when *N_th_* = 5. Being estimated for samples with a single BCC segment, these performance characteristics are overly pessimistic since BCC tumours typically spread over several segments in the AF image. For example, infiltrative BCCs typically consist of tens of microscopic (50-200μm) tumour regions. Thus, if we considered a more realistic case where the BCC is present in 10 segments, using *N_th_* = 5 leads to the per sample sensitivity and specificity of 99.93% and 95.65%, respectively.

Note that any sample containing non-BCC segments can have amongst its *N_BCC_* detections false alarms. Thus, even if such a sample contains BCC segments and is diagnosed to be “BCC-positive” *(N_BCC_*≥*N_th_),* this does not mean that all of its *N_BCC_* detections, nor even *N_th_* of them, are necessarily true positive. In the extreme case a BCC sample can be diagnosed as BCC while all of its *N_BCC_* detections are false positives. In practice, however, a sensible statistical test will need to assure that the chance of this happening is negligible. Using our test on samples with a single BCC segment, however, this probability (of correctly identifying such a sample as BCC based entirely on false positive segments) is 15.40% when *N_th_* = 1, but reduces to 0.78% when *N_th_* = 5. When a sample with more than 10 BCC segments, the probability drops below 10^−6^.

It is important to realise that all of the above calculations are based on simplifying assumptions, such as that of statistical independence of test outcomes from different segments, constant number of spectra per segment (and subsequently constant per segment performance characteristics). Thus, the above predictions may be overestimates of the real performance. Nevertheless, the above calculations provide a simple guidance for selecting the threshold *N_th_* in order to allow an objective way of establishing the diagnosis based on an MSH image. Further improvement of performance may be achieved by allowing the threshold *N_th_* to depend on additional parameters of the sample, including those which have been assumed to be constant in the above discussion.

### 3.2 Transferability of the algorithms on the automated MSH Prototype using independent samples

After the optimisation of the MSH algorithms using the training set of samples measured on the Laboratory instrument, the algorithms were implemented on the fully automated Prototype. A set of 47 independent skin samples (of which 32 BCC positive) from 40 patients undergoing Mohs micrographic surgery was used to evaluate the performance of each step of the algorithm in order to assess the inter-instrument transferability.

Figure 6(a)

**Fig. 6:**
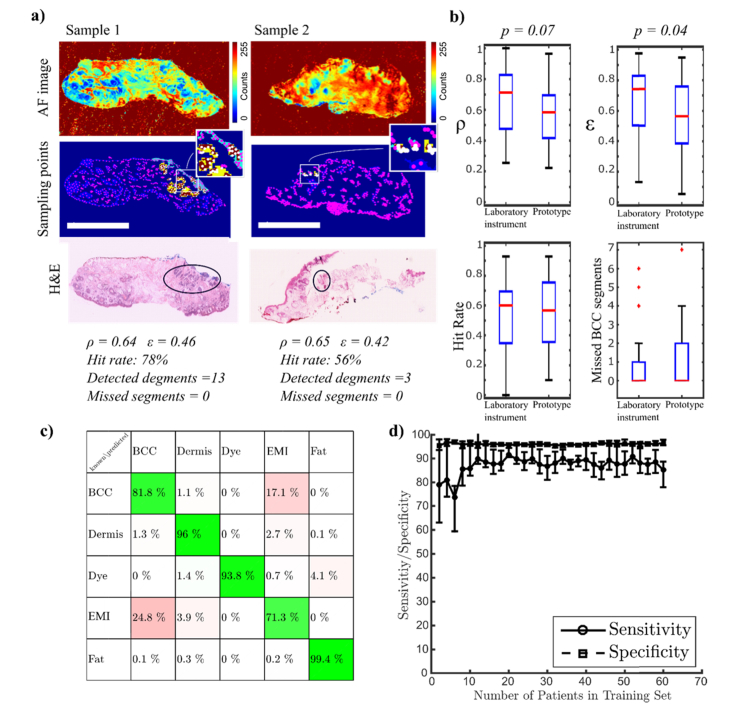
Transferability testing of the optimised models on the automated MSH Prototype. a) typical examples of AF images and segmentation results; Scale bars: 5mm. b) statistical analysis of the performance parameters ρ, ε, hit rate and number of BCC segments missed; c) confusion matrix of the Raman model applied on independent samples; d) 5-fold cross-validation of the joint training and test sets as a function of patient numbers.

presents the analysed AF images of two typical skin samples containing BCC. The tumour regions have low AF intensity, and are correctly captured by the segmentation algorithms (ρ= 0.64 and 0.65). The values of ε were 0.46 and 0.42, indicating that the segments containing BCC also contained ~60% healthy tissue.

Nevertheless, the “Hit rate” for BCC was 78% for Sample 1 and 56% for Sample 2, with no segments containing BCC being missed. The analysis of the performance metrics ρ and ε for all independent samples indicated a slight decrease compared to the values measured for the training set, though at the border of statistical significance (two-tailed t-test p-value of 0.07 and 0.04 respectively) (Fig. 6(b)). Nevertheless, the two parameters that influence directly the results of the MSH algorithm, the “Hit rate” and the number of missed BCC segments, are consistent with the training set. The median value of the “Hit rate” was 0.6 and the number of missed BCC segments per tissue sample ranged between 0 and 7 (0 median). The consistency of the results obtained for the independent test and training test indicate that the parameters related to AF image analysis optimised on the Laboratory instrument can be successfully transferred on the Prototype device. Figure 6(c) presents the confusion matrix for the independent test using the Raman classification model transferred on the Prototype instrument. The results confirm that BCC can be discriminated from dermis and fat with a 97.7% sensitivity and 99.3% specificity, which are similar to cross-validation results on the laboratory instrument. However, the sensitivity for discrimination of BCC and the EMI class (epidermis, inflammation and muscle) dropped to 71%, indicating that inter-instrument variations can mask subtle spectral differences between BCC and the EMI class.

Overall, the results indicate a sensitivity of 81.8%% and specificity of 92.5% for discriminating BCC from all other tissue types, an absolute 6% drop in both sensitivity and specificity compared to the 5-fold cross-validation results on the training set. After the independent test, the training and test data sets were merged and new 5-fold cross-validation analysis was performed as a function of the size of the training set (mixed Laboratory and Prototype instrument) (Fig. 6(d)).

### 3.3 Independent testing of MSH using the automated prototype instrument

After individual testing of the AF and Raman algorithms using independent samples, the performance of the MSH Prototype was tested using new independent samples. The MSH algorithm was based on Fig. 1(b) and contained the AF image analysis algorithms and Raman classification model trained using the samples measured on the Laboratory instrument.

**Fig. 7:**
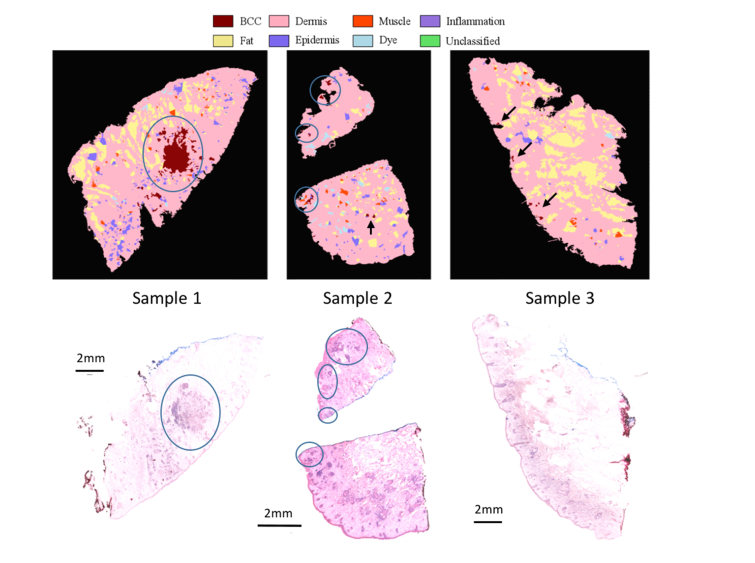
Comparison between MSH diagnosis (automated Prototype instrument) and the corresponding histopathology sections for typical skin layers excised during Mohs micrographic surgery. The diagnosis was based on the algorithm optimised on the training samples measured on the Laboratory Instrument. Sample 1 and 2 are BCC-positive and Sample 3 is BCC-negative. Tumours are encircled in blue circles and false positive segments in Sample 3 are highlighted by black arrows (circles and arrows were added manually).

Firstly, we tested the use of the threshold number *N_th_* = 5 that would allow a binary diagnosis of a skin sample as “BCC-positive” or “BCC-negative”. Sample 1 in Fig. 7

is an example of a skin sample with a large BCC of ~2mm. In this case, the number of segments labelled as BCC by the MSH algorithm was >20, which is higher than *N_th_*, and the sample was diagnosed “BCC-positive”. Sample 2 had 10 tumour regions, ranging between 100 μm to 1mm in size. Therefore, this sample is close to the detection limit of the instrument. The MSH analysis detected 8 BCC segments (6 true-positives and 2 false-positive segments), leading to the “BCC-positive” diagnosis. For the BCC-clear sample in Fig. 7 (Sample 3), the MSH diagnosis labelled 7 small segments as BCC. Because the number of segments labelled BCC is higher than the proposed threshold *N_th_* = 5, this sample was incorrectly diagnosed as “BCC-positive” (false-positive). This result indicates that the predicted 95.65% specificity for the *N_th_* = 5 threshold is likely to be an over-estimation, and suggests that the assumptions used in the calculations were too simplistic (i.e. ignore the correlation between the spectra in the samples). In addition, the calculations for the *N_th_* considered only the first round of Raman measurements. In the second round, additional Raman spectra are collected for segments that contained at least 2 but less than 80% spectra classified as BCC in the first round. If any of the Raman spectra in the second round are classified as BCC, but still do not reach the 80% threshold to label the whole segment as BCC, the initial segment is divided into smaller segment. Therefore, the second round of Raman measurements may lead to additional smaller false-positive segments in the final MSH image. This effect can be observed in Fig. 7: most false positive segments in Sample 3 are smaller in size than the true positive segments in Samples 1 and 2. To take into consideration these factors, a new threshold value was set at *N_th_* = 8. This new threshold would deliver the correct diagnosis for the samples presented in Fig. 7, and Sample 2 would represent a case at the limit of detection (8 segments labelled as BCC). Using the new threshold of *N_th_* = 8, our probabilistic model predicts to deliver 92.93% sensitivity and 99.93% specificity per tissue sample.

**Fig. 8:**
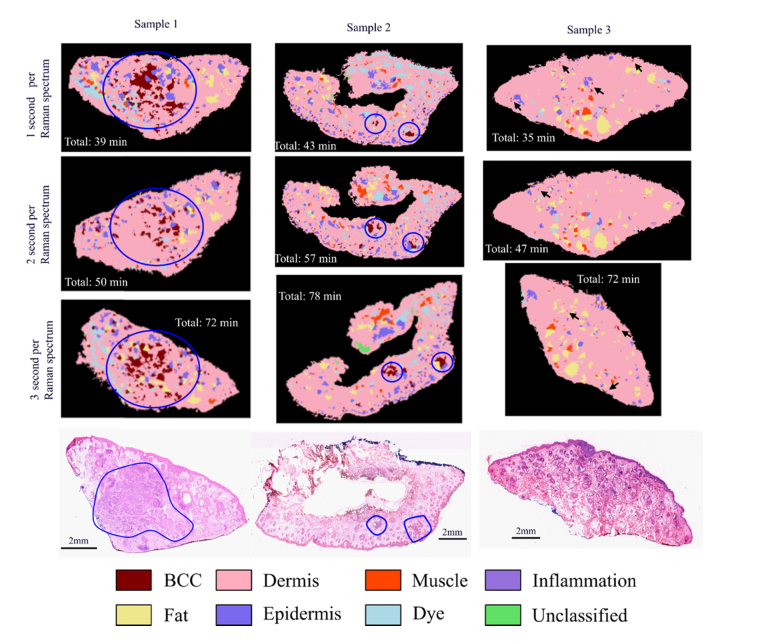
Consistency of MSH diagnosis using the automated Prototype instrument. Sample 1 and 2 are BCC-positive and Sample 3 is BCC negative. Tumours are encircled in blue circles and false positive segments in Sample 3 are highlighted by black arrows (circles and arrows were added manually).

Next, we evaluated the consistency of the MSH diagnosis by repeating the analysis three times on a set of three new samples (Fig. 8

). We included typical skin samples with large BCC (>4mm) and small BCC (e.g. 0.5-2 mm), and a BCC-clear sample. Each repeat measurement included the full analysis protocol: loading the tissue in the measurement cassette and loading the cassette into the instrument. At the end of the analysis, the cassette was unloaded from the instrument and the sample moved in a Petri dish. The stability of the Raman classifier was also evaluated by using different integration times for the Raman measurements (1s, 2s and 3s respectively). For Sample 1, >20 BCC segments were detected covering the area of the tumour, and 6-8 false positive segments were found at the edges of the sample (likely to correspond to inflamed dermis and hair follicles). Based on the *N_th_* = 8 threshold, correct BCC-positive diagnosis was obtained for all repeat measurements. Sample 2 represented a sample at the limit of detection as it contained only two main BCC regions. One region had a tumour of ~1mm and few additional microscopic tumours, while the second region had a main tumour of ~1.5mm surrounded by 12 microscopic tumours (size 100-200μm). Nevertheless, the sample was correctly diagnosed as BCC-positive in all repeat measurements because the MSH image detected >12 BCC segments.

All MSH images included >6 segments correctly classified as BCC, as well as 6-8 false positive segments, approximately 100-200μm in size. While the locations of the BCC segments were consistent for the three repeat measurements and in agreement with the histopathology slide, the false positive measurements were randomly distributed. For Sample 3 the numbers of false positive segments in the MSH images (6, 1 and 3 corresponding to the 1s, 2s and 3s integration time, respectively) were lower than the threshold *N_th_* = 8. Thus, MSH provided the correct “BCC-negative” diagnosis in all repeat measurements. Similar to the other samples, the false positive segments were randomly located in the images, and were approximately 10-20μm in size (only one false positive segment reached ~100μm).

The consistent diagnosis results confirm the high repeatability of the MSH diagnosis with respect to the process of tissue handling (loading/unloading of the tissue sample in the measurement cassette, loading/unloading of the cassette within the instrument) and insensitivity to positioning the tissue within the field of view of the instrument. When using *N_th_* = 8, correct diagnosis was obtained for all samples, even for the shortest integration time of 1s per spectrum. For this measurements, the total analysis time was 35-45 minutes, which is similar to frozen section histopathology. Increasing the integration time for the Raman measurements to 2s and 3s led to longer analysis times (47-57 minutes and 72-78 minutes, respectively) but decreased the number of false positive segments.

Finally, we evaluated the performance of the automated Prototype device when operated by non-specialist spectroscopy users. The MSH diagnosis obtained by two clinical users was compared to the result obtained by a spectroscopy specialist, as well as the reference histopathology. MSH is based on automated measurement and analysis algorithms, and the quantitative diagnosis is presented in a colour-coded image, in which each colour corresponds to a tissue class. The final diagnosis at a tissue level is obtained by comparing the number of BCC classified segments in the MSH image with a threshold value *N_th_*. Because the actual tissue analysis is user-independent, the user requires training only on tissue handling (few hours). To demonstrate this feature, three users carried out repeat MSH analysis on a new set of skin specimens excised during Mohs micrographic surgery. User 1 (R. Boitor) was specialist in Raman spectroscopy and was the main person developing the MSH algorithms. User 2 (S. Varma) was a Mohs surgeon, and User 3 (B. Salence) was a core medical trainee intending to pursue a speciality in dermatology.

User 2 and 3 had no experience in Raman spectroscopy and had ~1 hour, respectively 8 hours, training on tissue handling (load/unload tissue in cassette) and operation of the prototype instrument (load cassette in the instrument, input patient data, and start/stop analysis).

Figure 9

**Fig. 9:**
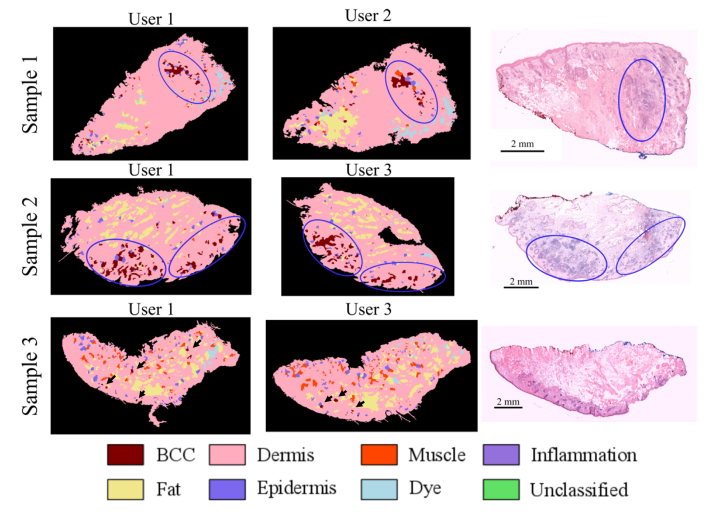
Consistency of MSH diagnosis among different users. User 1: spectroscopy specialist (R. Boitor), User 2: Mohs surgeon (S. Varma) with 1 h training, User 3 (B. Salence): core medical trainee with interest in dermatology (BS) with 8 hours training. Tumours are encircled in blue circles and false positive segments in Sample 3 are highlighted by black arrows (circles and arrows were added manually).

presents the MSH results obtained by the three users, and compares the results with the reference histopathology slides. For the BCC-positive samples (Samples 1 and 2), the number of segments classified as BCC was higher than the threshold value *N_th_* = 8 for all measurements, leading to correct “BCC-positive” diagnosis. For the BCC clear sample (Sample 3) analysed independently by User 1 and User 3, the number of false positive segments was 7 and 5, respectively, indicating consistent “BCC-negative” diagnosis. These results highlight the advantage of MSH diagnosis in providing a reliable and quantitative diagnosis, even when the analysis was carried out by non-specialist users with training as short as 1 hour. This feature is important when considering the deployment of the technology in the clinic; non-specialist users can operate the devices and obtain valid and repeatable diagnosis of each resected tissue layer in order to confirm the completeness of tumour excision. This is a key advantage compared to conventional histopathology or alternative optical microscopy techniques based on structural imaging, where users require extensive training and experience in order to recognise specific structural and morphological features required for diagnosis.

## 4. Conclusions

In this paper we present a fully automated multimodal spectral histopathology (MSH) Prototype instrument for detection of residual tumor during surgery of basal cell carcinoma (BCC). The instrument has been designed to be used in a clinical environment and be operated by non-specialist users. First, the data acquisition and analysis algorithms were optimized on a manually-operated Laboratory instrument, and then successfully transferred on the automated Prototype. We demonstrate that accurate diagnosis of residual BCC can be obtained in an automated manner on a range of skin specimens excised during Mohs micrographic surgery. Typical acquisition times for tissue samples with areas of 2cm x 2cm was less than 60 minutes, allowing detection of tumors as small as 100 μm. Using a simple binary diagnosis protocol based on a threshold number of BCC detected segments in the MSH images (*N_th_* = 8), the correct diagnosis was obtained for all samples, in three repeat measurements (confirmed by histopathology as the standard of reference). We also show that the instrument can be operated by non-specialist spectroscopy users, including one Mohs surgeon and one core medical trainee, after training in tissue handling and instrument operation of 1-8 hours. The MSH diagnosis obtained by the non-specialist users was consistent with the results obtained by the spectroscopy specialist user, and agreed with the diagnosis provided by histopathology. This is an important result considering that the intended users of the device are histopathology technicians, nurses or dermatology surgeons. This is the first fully-automated prototype instrument based on Raman spectroscopy for intra-operative microscopic imaging of surgical margins during cancer surgery, suitable to be used by a non-specialist user in a clinical environment. The development of the prototype is an important step on the clinical translation path, as it will allow the testing of the multimodal spectral histopathology technique in a relevant clinical environment in order to evaluate its performance on a sufficiently large number of patients.
